# Male grey seal commits fatal sexual interaction with adult female harbour seals in the German Wadden Sea

**DOI:** 10.1038/s41598-020-69986-w

**Published:** 2020-08-13

**Authors:** Simon Rohner, Kirsten Hülskötter, Stephanie Gross, Peter Wohlsein, Amir Abdulmawjood, Madeleine Plötz, Jutta Verspohl, Ludwig Haas, Ursula Siebert

**Affiliations:** 1 Institute for Terrestrial and Aquatic Wildlife Research, University of Veterinary Medicine Hannover, Foundation, Büsum, Germany; 2 Department of Pathology, University of Veterinary Medicine Hannover, Foundation, Hannover, Germany; 3 Institute for Food Quality and Food Safety, Research Center for Emerging Infections and Zoonoses, University of Veterinary Medicine Hannover, Foundation, Hannover, Germany; 4 Institute for Microbiology, University of Veterinary Medicine Hannover, Foundation, Hannover, Germany; 5 Institute of Virology, Department of Infectious Diseases, University of Veterinary Medicine Hannover, Foundation, Hannover, Germany

**Keywords:** Ecology, Genetics, Ocean sciences, Pathogenesis

## Abstract

Males of several seal species are known to show aggressive copulating behaviour, which can lead to injuries to or suffocation of females. In the North Sea, grey seal predation on harbour seals including sexual harassment is documented and represents violent interspecific interaction. In this case series, we report pathological and molecular/genetic findings of 11 adult female harbour seals which were found dead in Schleswig–Holstein, Germany, within 41 days. Several organs of all animals showed haemorrhages and high loads of bacteria, indicating their septic spread. All females were pregnant or had recently been pregnant. Abortion was confirmed in three cases. Lacerations were seen in the uterus and vagina in six cases, in which histology of three individuals revealed severe suppurative inflammation with intralesional spermatozoa. Molecular analysis of vaginal swabs and paraffin-embedded samples of the vagina identified grey seal DNA, suggesting violent interspecific sexual interaction with fatal outcome due to septicaemia. This is the first report of female harbour seals dying after coercive copulation by a male grey seal in the Wadden Sea.

## Introduction

There are three main forms of how males sexually coerce females in animal societies: intimidation (punishment for denial of mating), harassment (increase of costs for females if they refuse to mate) and forced copulation (use of superior speed or physical strength to copulate with the female by force)^1^. Sexual coercion is widely distributed among animal clades and seen in birds^2,3^, mammals^4,5^ and invertebrates^6,7^. Coercive behaviour of males reflects the competition for limited resources, in this case fertile females^8^. It may be more common among species with polygynous mating systems^9^, including pinnipeds, where the dominance over a harem results in a substantial breeding reward for the male^10-12^. Harassment and mating attempts can result in injuries to the females, such as bite wounds, mostly in the region of the face and the neck^12^. Suffocation by being crushed by substantially larger males may even lead to death, as witnessed in New Zealand sea lions (*Phocarctos hookeri*)^13^. Such aggression seen in adult male pinnipeds is usually focused on conspecifics and are mainly displayed in land breeding species like otariids and some phocids including grey seals *(Halichoerus grypus)* and elephant seals (*Mirounga *spp*.*)^14^.

Hybrids as the result of interspecific mating among sympatric species are not uncommon in the animal kingdom^15,16^. Anatomical features and behavioural aspects including mate choice and reproductive competition may represent important limitations for such interspecies events^15,16^. In most species, the female choice of a mating partner is considered to be a main factor to avoid hybridisation, reflecting their higher fitness and reproductive costs^16^. In marine mammal polygynous mating systems though, the male dominates the mate choice, whereas the female contribution can be regarded as minor^17^. Behavioural indicators for female receptivity in marine mammals are often limited to reduced aggression and increased acceptance during mating attempts^12^. Whereas experienced males rarely provoke female cooperation more than once, inexperienced males may not read the female body language properly^12^. It can be assumed that especially juvenile, rather inexperienced male seals without access to conspecific, receptive females try to force copulation with juveniles or females of other species, they can overpower^18-21^. In these cases, male intromission and ejaculation often fail due to anatomical or behavioural disparities^11,20,22^. Nonetheless, some reports of hybrids among pinnipeds in the wild and in zoological enclosures exist^23–25^.

Harbour seals (*Phoca vitulina*) and grey seals share habitats in the North Sea and there is evidence of direct competition between the two apex predators for certain resources^26,27^. Harbour seals tend to inhabit coastal waters more exclusively^28^, whereas grey seals also forage offshore to a large extent^29^. Haul-out sites and foraging areas can overlap with regional differences^27,30^. Whereas the number of grey seals in German waters is still increasing through recolonisation after local declines in the past (6,538 counted in 2019)^31,32^, the number of harbour seals remains stable (27,763 counted in 2019)^32,33^. Mass mortalities caused by phocine morbillivirus and avian influenza virus (H10N7) had detrimental effects on the harbour seal population^34-36^. The grey seal population seemed to be less affected, although it may have functioned as a vector for these pathogens^37,38^. Septicaemia with β-haemolytic streptococci and/ or *E. coli* is an important cause of death of adult harbour seals from German waters, which in many cases is the result of major pathologies such as gastritis, intestinal volvulus or mastitis. Active cannibalism among grey seals, also in the North Sea, has been previously reported^39-42^. Predation on other marine mammals, including harbour seals, adds to this aggressive behaviour and might represent a feeding strategy^43-45^.

Forced mating attempts and even successful copulations between male grey seals and harbour seals have been observed, albeit it is not reported whether or not the affected victims suffered from injuries or even died^21,46^. Young adult male grey seals have been witnessed to engage in interspecific mating attempts on the Island of Helgoland in the German North Sea, but related genital injuries or abortion were not documented^43^. This case series from the Wadden Sea for the first time describes morphological and molecular findings in pregnant female harbour seals after copulation with a male grey seal with subsequent fatal septicaemia, likely due to genital lacerations.

## Results

**Figure 1:**
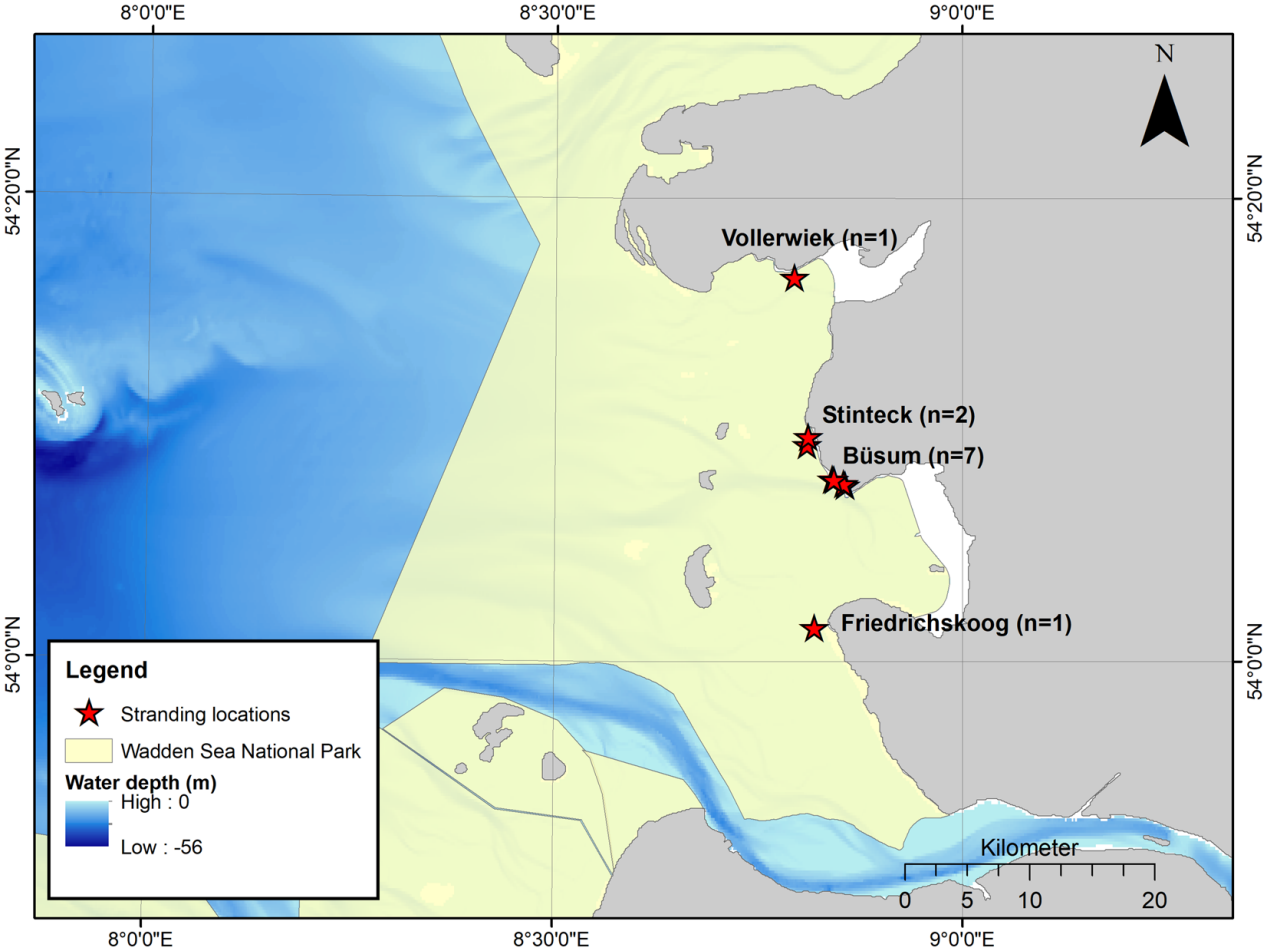
Harbour seal strandings between December 2019 and January 2019 along the western shore of Dithmarschen, Schleswig–Holstein, Germany. Map made with ArcGIS for Desktop version 10.5 (ESRI, Inc., USA) (https://www.esri.com), using the country shapefile (GSHHG, version 2.3.7, 2017). Information contained here has been derived from data that is made available under the European Marine Observation Data Network (EMODnet) Seabed Habitats initiative (https://www.emodnet-seabedhabitats.eu/), financed by the European Union under Regulation (EU) No 508/2014 of the European Parliament and of the Council of 15 May 2014 on the European Maritime and Fisheries Fund.

**Figure 2:**
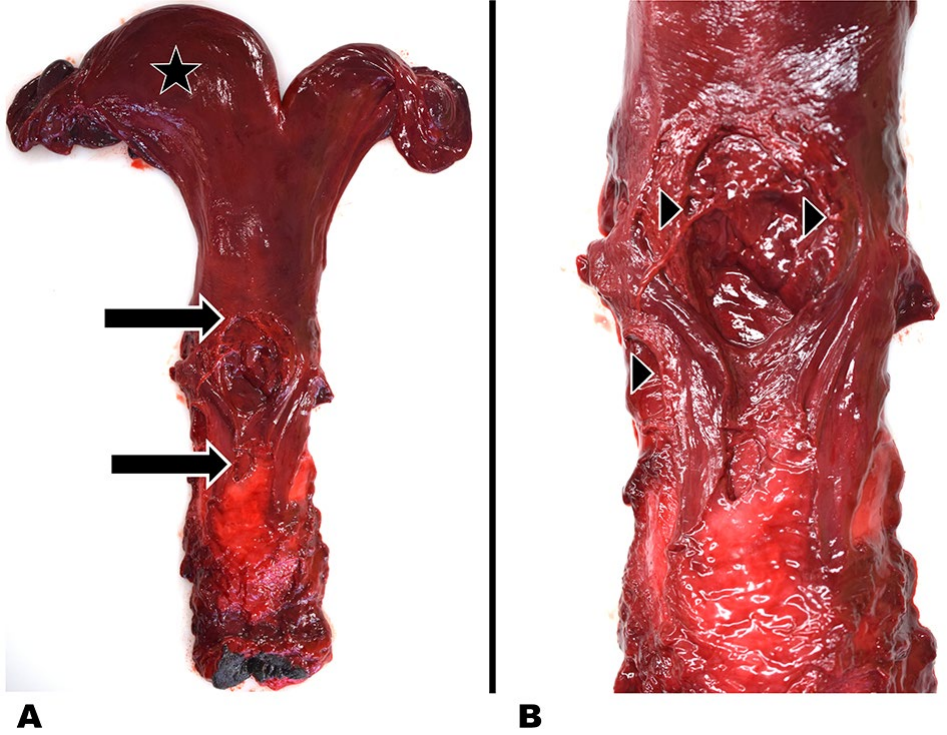
Uterine lacerations of harbour seal (*Phoca vitulina*) no. 9. The left uterine horn is enlarged (black star) (**A**), dorsally located defect of the *corpus uteri* (**A**) (black arrows) with a step-like margin (black triangles) and attachment of fibrin to the serosa (**B**).

**Figure 3:**
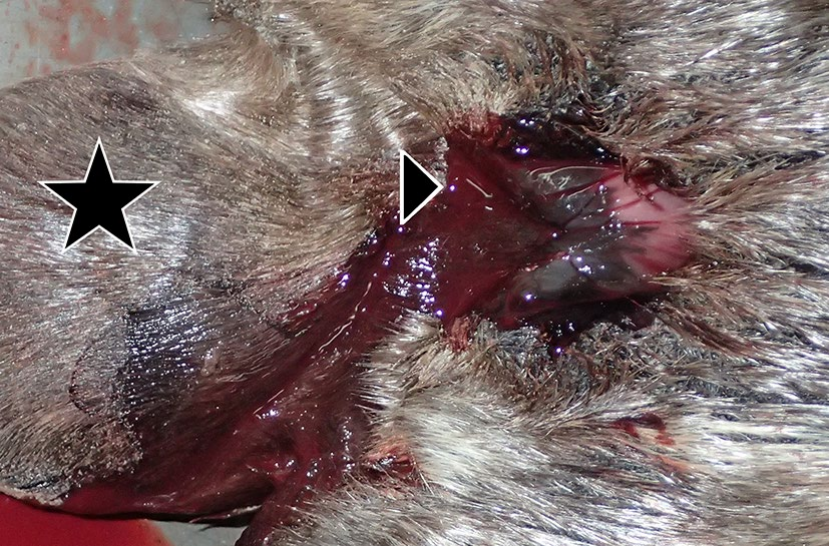
Haemorrhagical discharge from the vulva (black triangle) of harbour seal (*Phoca vitulina*) no. 11. The tail is pictured with a black star.

**Figure 4:**
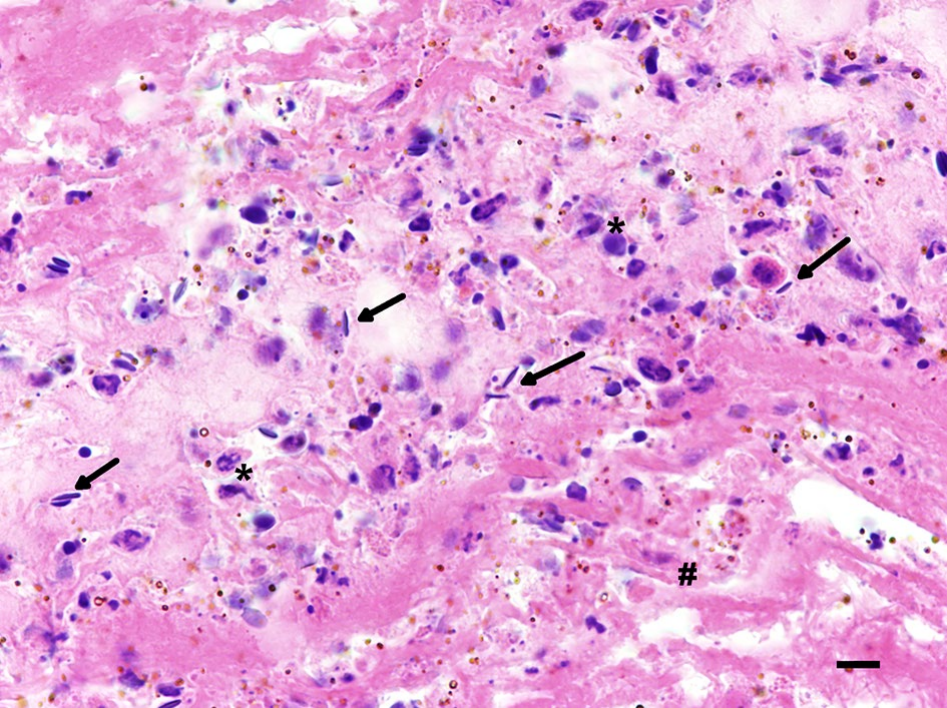
Histological specimen of the vagina (no. 11) with infiltration of inflammatory cells (*) and spermatozoal heads ( →) within a meshwork of fibrin (#). HE-stain. Bar = 10 µm.

Eleven harbour seal strandings occurred between 4 December 2018 and 13 January 2019 along a confined part of the western coastline of Dithmarschen, Schleswig–Holstein, Germany (Fig. 1). All seals (nos. 1–11) were adult females in a good nutritional status (Table 1). Six animals were fresh, five in a state of moderate decomposition. Signs of scavenging occurred around the head and eyes of four and in the anal and genital region of three seals. Genital lacerations were found in six animals ranging from 1 to 12 cm in length. Four individuals (nos. 5, 6, 8, 10) had lacerations in the vagina, one (no. 11) had lacerations in its vagina and uterus, and in one individual (no. 9), only a uterine lesion was present (Fig. 2). The lacerations were located dorsally (nos. 6, 8, 9, 10, 11) in the vagina and/or uterus, and only one was detected in the ventral aspect of the vagina (no. 5). Tissue fissures were longitudinal with a step-like appearance with the largest defect seen in the serosa, followed by the muscular layer, and the smallest defect within the mucosal layer, indicating a protracted tearing of the tissue layers according to their individual elasticity. The margins were irregular with multiple tissue bands and partly of filamentous appearance due to torn muscle fibres (Fig. 2). Mostly mild fibrin extravasation gave the serosa a coarse surface (Fig. 2). Compared to livestock and other terrestrial mammals, the tissues of marine mammals show a significantly darker red staining, reflecting their increased haemoglobin and myoglobin concentrations and oxygen storage capacity^47^. Therefore, macroscopically dark red colourations due to haemorrhages do not vary much compared to the unscathed uterine or vaginal tissue and need to be confirmed histologically. In three cases (nos. 8, 9, 11), a swollen vulva with serous, red discharge was observed (Fig. 3). Histology revealed severe fibrinous and suppurative inflammation with infiltration of neutrophils and a few macrophages adjacent to the genital lesions in two females (nos. 9, 11). In one case, there was more prominent extravasation of proteinaceous fluid, minor mononuclear infiltration and oedema (no. 8). Within the proteinaceous fluid and fibrin, accumulations of bacteria and spermatozoa-like structures were detected in these three cases (nos. 8, 9, 11) (Fig. 4).

**Table 1:** General information and post-mortem findings in the reproductive organs of 11 stranded female harbour seals (Pv).

Case no.	Stranding date	Locality	Vagina	Uterus	Foetus	Abortion
Pv 1	04.12.2018	Büsum	No detectable lesions	Left horn enlarged	Cervix; 11,1 cm length	Yes
Pv 2	05.12.2018	Büsum	No detectable lesions	Right horn enlarged	Right horn; 19 cm length	No
Pv 3	08.12.2018	Büsum	No detectable lesions	Right horn enlarged	None	Suspected (see Uterus)
Pv 4	09.12.2018	Büsum	No detectable lesions	Left horn enlarged	None	Suspected (see Uterus)
Pv 5	09.12.2018	Westerdeichstrich	Laceration ventrally	Right horn enlarged	Abdomen; fragments	Yes
Pv 6	09.12.2018	Büsum	Laceration dorsally	Right horn enlarged	None	Suspected (see Uterus)
Pv 7	10.12.2018	Vollerwiek	No detectable lesions	Left horn enlarged	Left horn; 20 cm length	No
Pv 8	11.12.2018	Büsum	Laceration dorsally	Left horn enlarged	Left horn; 16 cm length	No
Pv 9	30.12.2018	Stinteck	No detectable lesions	Left horn enlarged; Lacerations dorsally	None	Suspected (see Uterus)
Pv 10	07.01.2019	Friedrichskoog	Laceration dorsally	Left horn enlarged	None	Suspected (see Uterus)
Pv 11	13.01.2019	Büsum	Small laceration dorsally	Right horn enlarged; laceration dorsally	Abdomen; 32 cm length	Yes

Six females (nos. 1, 2, 5, 7, 8, 11) were pregnant carrying a foetus. Five seals had been recently pregnant, unilaterally showing an enlarged uterine horn (nos. 3, 4, 6, 9, 10). Additionally, four of these individuals showed tissue and/or a creamy, partly clear and partly red substance in the enlarged uterine horn, indicating remaining placental tissue and amniotic fluid (nos. 3, 4, 6, 9). One foetus (total length 32 cm) was displaced into the abdominal cavity associated with a rupture of the uterine wall (no. 11). Another foetus was stuck in the cervix (no. 1). The total length of the foetus was 11.1 cm. Bones with cartilage, together with brownish foul-smelling fluid, the remains of a decomposed foetus, were present within the abdominal cavity of a third female with a vaginal defect (no. 5). Macroscopically as well as histologically, general hyperaemia was most pronounced in the lungs, brains and kidneys, accompanied by severe haemorrhages in the lungs, intestines and brains. The spleen was swollen with bulging dark red follicular structures on the cut surface in nine cases, indicating hyperplasia of the red pulp (nos. 1–7, 9, 11). Furthermore, the mesenteric lymph nodes were hyperplastic in eight animals (nos. 1, 2, 3, 5, 7, 8, 10), implying an immunological activation. Several mesenteric and pulmonic lymph nodes were hyperaemic and showed resorption of blood (nos. 3, 4, 6, 7, 9, 10).

Ultrastructural analysis of vaginal tissue samples, with spermatozoa-like structures seen histologically, revealed numerous elongated spermatozoal heads with a heterochromatic nuclei (3.63 × 0.68 µm) ensheathed by acrosomes. Spermatozoal heads were largely disconnected from the middle pieces and flagellum. The preservation of the mitochondria in the middle pieces was largely indistinct, which reflected the beginning of autolysis. In cross sections, the middle pieces showed remnants of mitochondria surrounding the central axonemal microtubules and the outer dense fibres. Flagella showed a pair of central axonemal microtubules surrounded by nine, typically arranged microtubules (9 + 2). While there were still outer dense fibres in the principal pieces of the flagellum, these were absent in the distal parts.

**Figure 5:**
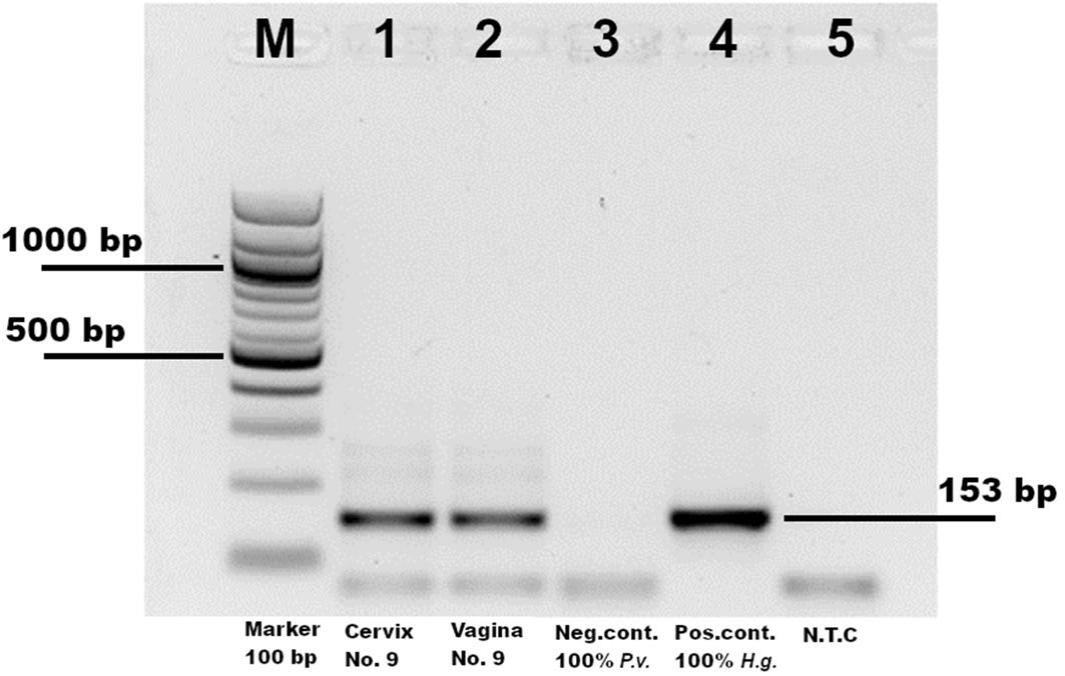
Typical amplicon of *halichoerus grypus*-specific-PCR, ‘M’ DNA marker 100 ladder. Lane 1 demonstrates the MSwab samples of the cervix, Lane 2 MSwab samples of the vagina of seal no. 9, both of them positive, Lane 3 shows the negative control (*Phoca vitulina*), Lane 4 Lane show the positive control (*Halichoerus grypus*) and Lane 5 non-template control.

DNA-extraction and PCR of five formalin-fixed, paraffin-embedded (FFPE) genital tract specimens of the three animals with histologically visible spermatozoa (nos. 8, 9, 11) revealed grey seal DNA in all samples (Fig. 5). All control samples of harbour seals, including non-reproductive tract organ samples of harassed animals, as well as the reference samples of the seals, were negative for grey seal DNA.

Microbiology revealed mild to severe growth of *Escherichia coli* and β-haemolytic streptococci in the uterus, lung, liver, kidney, spleen and brain of all seals (Table 2).

**Table 2:** β-haemolytic Streptococci and *Escherichia coli* in the main affected organs of 11 stranded female harbour seals (Pv).

Case no.	Lung	Liver	Kidney	Spleen	Uterus	Brain
*β-haemolytic Streptococci*						
Pv 1	Severe	Severe	Severe	Severe	Severe	Severe
Pv 2	Moderate	Moderate	Moderate	Moderate	Moderate	Moderate
Pv 3	Severe	Moderate	Severe	Severe	Moderate	Moderate
Pv 4	Severe	Moderate	/	Severe	Moderate—severe	Moderate
Pv 5	Moderate	Severe	Moderate	Severe	Severe	Mild-moderate
Pv 6	Mild-severe	Mild-severe	Severe	Mild-severe	Severe	Moderate
Pv 7	Mild	Severe	Moderate	Moderate	Severe	Mild
Pv 8	Moderate	Moderate	Mild	Moderate	Moderate	Mild
Pv 9	Moerate—Severe	Mild	Severe	Severe	Severe	Severe
Pv 10	Moderate	/	/	/	Severe	Moderate—severe
Pv 11	Mdoerate—severe	Moderate	Severe	Severe	Severe	Moderate
*E. Coli*						
Case no.	Lung	Liver	Kidney	Spleen	Uterus	Brain
Pv 1	Severe	Severe	Severe	Severe	Severe	Severe
Pv 2	Mild	Moderate	Mild	Mild	Moderate	Mild
Pv 3	Severe	Severe	Severe	Severe	Moderate	Moderate
Pv 4	Severe	Moderate	/	Moderate	Severe	Severe
Pv 5	Moderate	Severe	Moderate	Moderate	Severe	Moderate
Pv 6	Severe	Moderate	Moderate	Severe	Severe	Moderate
Pv 7	Mild	Mild	/	/	Severe	Mild
Pv 8	/	Mild	Mild	Severe	Moderate	/
Pv 9	Moderate	Mild	Mild	Mild	Moderate	Severe
Pv 10	Severe	Moderate	/	/	Mild	Severe
Pv 11	Moderate	Moderate	Mild	Mild	Moderate	Moderate

Virological analyses for Phocine Distemper Virus (PDV), Phocine Herpes Virus (PHV) and Influenza Virus were negative in all seals.

## Discussion

The presented fatal cases of 11 adult, female harbour seals could be traced back to interspecific copulation with a grey seal bull via detection of spermatozoal structures in the female reproductive tracts as well as grey seal DNA verification. Pathological changes seen in the genital tracts included haemorrhagic or suppurative vaginal discharge, genital lacerations and abortion. Death occurred due to septicaemia with β-haemolytic streptococci. The narrow period, within which the strandings happened, coincided with the breeding season of grey seals in the North Sea. As such incidences have not been reported before in the Wadden Sea area, they provide new insights into interspecific interactions between the two native seal species.

The majority of annually recovered seals by the German stranding scheme are juveniles^34^. Previous instances of unusually large numbers of adult mortalities were exclusively seen during the epizootic events caused by influenza and morbillivirus^34–36^. However, this was excluded in the present case as the virological tests were all negative.

Furthermore, it is exceptional that only females were affected that were either pregnant or at least demonstrated a recent pregnancy. This fact also doubles the death toll to potentially 22 animals in the context of this event. In contrast to the winter breeding season of grey seals, the pupping season of harbour seals in the Wadden Sea starts in June^48^. Both, the pregnancy as well as the season allows the assumption that the female harbour seals themselves were not interested in mating at this point.

Along the coastline of Dithmarschen, there are several sandbanks close to the shore, where harbour seals haul out routinely and grey seals are known to visit these occasionally. Seals are generally polygamous and depending on the group size, the sex ratio and the social system, different aggressive mating strategies can develop^53,56–59^. Injuries during interspecific interactions become more likely if there is great disparity in size and weight^52^. This is also reported for the here involved species, as a juvenile or young adult grey seal bull can easily over-power and subdue a female harbour seal^25,26,60^. The behavioural response of the female during mating is important for the stimulation of the male. If the female is incompatible, juvenile, seriously injured, or dead, the response may be inadequate and could result in multiple mountings and/ or longer mating attempts^11,19^. In the presented cases, vaginal discharge and a swelling of the vulva in some cases were the only external lesions.

Microbiological investigations of the 11 female carcasses revealed synergistic β-haemolytic streptococci infections together with *E. coli* in various organs. It is known that the changed immune response and physiology during pregnancy may facilitate septic infections with fatal progression^49^. This also might have been a driving co-factor of the presented cases. In pinnipeds, β-haemolytic streptococci are commensal bacteria and range among the most frequently detected pathogens regarding secondary septic infections^50,51^. A mass die-off in cape fur seals (*Arctocephallus pusillus*) including abortions in high numbers was attributed to starvation, high parasite burdens and following secondary infections with β-haemolytic streptococci^52^. Similarly, in German waters, high parasite loads and associated morphological changes in the respiratory or digestive tract often lead to secondary septicaemia in seals^34^. In contrast to this, the herein reported harbour seals were all in good nutritional status with low parasite burdens. Furthermore, a retrospective data analysis revealed that from 2015–2020, nine out of 24 adult female harbour seals retrieved from the German North Sea died due to septicaemia with *β-haemolytic streptococci* and *Escherichia coli*. In four cases, the animals had suffered from serious alterations of mainly the gastrointestinal tract, leading to the septic spread of these opportunistic bacteria. Of the 11 female harbour seals, six showed vaginal and/ or uterine lacerations, where the bacteria could have entered the bloodstream. Additionally, superficial lesions (erosions and ulcerations) can disrupt the epithelial barrier function of the vagina and facilitate the entry of opportunistic pathogens leading to infection. The haemorrhagic discharge and microscopically verified vaginitis underline an intra vital vaginal defect and infection in the six animals. However, macroscopically, lacerations or inflammation of the genital tract could not be documented in all seals.

Histological analysis verified hyperaemia and haemorrhages in several organs as typical findings of septicaemia. As there were no signs of either haematoma in soft tissues or bone or cartilage fractures, the contribution of blunt trauma and/ or compression to the haemorrhages cannot be finally clarified. Furthermore, fibrino-suppurative inflammation in the reproductive tracts with intralesional spermatozoal heads were found in three out of six sampled animals. The embedding of spermatozoa within the inflammatory exudate is an indicator of intra vital copulation. Using transmission electron microscopy, the detached tails of the spermatozoa were depictable within a meshwork of extravasated fibrin (see supplementary materials, Fig. 1). The disintegration of spermatozoal structures reflects autolytic and heterolytic processes. Still, it is not possible to use the decomposition status of the spermatozoa to suggest a specific timeframe of copulation. The genital lacerations point towards traumatic copulation and to an assailant larger than a harbour seal. All formalin-fixed and paraffin-embedded (FFPE) tissues of harbour seal reproductive tracts with histological spermatozoa were tested via PCR and contained grey seal DNA. FFPE samples of other organs of the affected animals, as well as unrelated cases retrieved from the local archive were used as control samples to exclude false positive signals due to contamination and did not contain grey seal DNA. Environmental contamination of the harbour seal reproductive tracts with grey seal DNA to an extent causing a positive PCR reaction still seems unlikely. Therefore, it can be assumed that the spermatozoa within the samples are of direct grey seal origin. As stated before, male intromission and ejaculation in interspecific mating attempts is not always successful due to anatomical and behavioural incompatibility^11,20,22,53^. Accordingly, intralesional sperm is not an obligatory finding in suspected cases of interspecific mating, but it can be critical for identifying the assailant.

There are reports of necrophilia in marine mammals and other clades^11,19,53,54^. In the reported cases with intralesional spermatozoa, an inflammatory response with extravasation of proteinaceous fluid and fibrin, engulfing the spermatozoal heads, underlined an intra vital process. Still, it cannot be excluded that tissue defects without haemorrhage or signs of inflammation are the result of post-mortem mountings. Feeding on victims that died after violent inter- or intraspecific encounters is not reported in other seal species^11,20^. This might be different in grey seals, as coercive behaviour and predation are sometimes displayed simultaneously^43^.

Most reports of mating-related trauma in pinnipeds refer to external, dorsal head and neck wounds of females after being bitten, pinned down or dragged by males^13,55–57^. Whereas likewise observations for aggressive mating behaviour and external lesions exist for many other vertebrate classes, evidence of internal injuries remains scarce^55,58^. A well-documented case was reported in several male southern sea otters (*Enhydra lutris nereis*) force-copulating with juvenile Pacific harbour seals (*Phoca vitulina richardsi*)^53^. Necropsies in eight cases revealed vaginal and colorectal perforations of 1–3 cm in size and even a vaginal-cervical translocation in one case. The perforations seen in the vagina were typically located on the dorsal and lateral side. As a result of the lesions, the animals died after developing peritonitis and sepsis. Five out of six lacerations seen in the reproductive tracts of the 11 female harbour seals were also located dorsally in the vagina and/ or uterus. Together with the septic infections caused by β-haemolytic streptococci, there are clear analogies to the reported cases in sea otters.

The lacerations of the uterus in two of the presented cases, as well as the dislocation of the foetus into the abdomen could not be explained by penile penetration alone, although the vaginal anatomy of harbour seals indicates that the depth of penetration depends only on the length of the penis^22^. In the case of a male elephant seal (*Mirounga leonina*) mounting female cape fur seals (*Arctocephalus pusillus*), it was observed that the weight of the male forced out the females intestines through the anogenital region^20^. Even a juvenile male grey seal can outweigh a female harbour seal^28,29^, but it remains unclear whether the mounting of pregnant females alone can lead to lacerations in the reproductive tract. Rupture of the gravid uterus is reported to be a rare event, and is often associated with severe abdominal trauma or fragility of the uterine wall due to infections or previous operations^59^. In the reported cases, it cannot be excluded that ascending infections or sepsis led to structural weakness of the uterus with secondary rupture. Since there were no observations of the encounters in the described cases, the extent of violence during the mating attempts and their possible contribution to the genital trauma, as seen in other cases^11,19^, cannot be assessed.

Grey seals are known to engage in inter- and intraspecific interactions, including predation^43^, cannibalism^39–42^ and forced sexual intercourse^21^ with harbour seals in the wild. The present study is the first one reporting fatal interspecific mating attempts of at least one male grey seal with pregnant harbour seals in the German Wadden Sea. It remains unclear if the described cases are an exception caused by one individual on a local scale. Therefore, future post-mortem investigations should always include a precise examination of genital tracts of harbour seals especially during the grey seal mating season.

## Conclusions

Besides predation, sexual coercion appears to be another phenomenon by which grey seals negatively impact harbour seals. Apparently, especially juvenile males without access to fertile females display such behaviour. Due to missing or failing defensive behaviour, disparity in size and aggressive mating strategies, these encounters can be fatal for female harbour seals. The presented cases show that pathological indicators of sexual coercion in harbour seals may be haemorrhagic or suppurative vaginal discharge, genital lacerations and abortion. Spermatozoa may not always be detectable, but can serve as a valuable indicator of lesion genesis and can help in identifying interspecific encounters.

## Materials and methods

The 11 adult female harbour seals found dead along the coastline of the German federal state of Schleswig–Holstein between 4 December 2018 and 13 January 2019 were collected through the stranding network of Schleswig–Holstein. Necropsies were performed on all animals following a standardised protocol, either fresh or for organisational reasons after being frozen^34^. Lacerations and wound margins in the reproductive tracts were documented and measured. The reproductive tracts of two animals were stored frozen as a back-up sample and later used for detecting grey seal DNA. Various tissues of all seals, including the different sections of the reproductive tract (vagina, cervix, and uterus) of six animals, were sampled for histopathological analysis. The samples were fixed for 24 h in 10% buffered formalin, embedded in paraffin wax and 2–3 µm thin sections were cut and stained with haematoxylin and eosin (HE) in accordance with a routine protocol^34^. Parasites were collected during the necropsies, stored in 70% ethanol and were identified microscopically^60^.

Detection of PDV, PHV and Influenza virus was performed via polymerase chain reaction (PCR) and reverse transcription polymerase chain reaction (RT-PCR) using different tissue samples and throat swabs^36,61^.

For cultural examination, organs with the exception of intestinal samples were plated on Columbia agar with sheep blood (Thermo Scientific, Applied Biosystems, Invitrogen, Gibco or Ion Torrent), Gassner agar (Thermo Scientific), Staph/Strep selective medium (in-house) and placed in nutrient enrichment broth (in-house). The cultures were incubated over night at 37 °C. On the following day, the nutrient broth was isolated onto the same media as mentioned above and incubated until the following day. Intestinal samples were cultured on Columbia agar with sheep blood and Gassner agar. The swab was then washed out in two Salmonella selective enrichment broths, tetrathionate brilliant green bile broth (TBG; VWR) and Rappaport Vassiliadis Soya broth (RVS; Thermo Scientific). Cultures were incubated at 37 °C overnight. On the following day, the liquid media were streaked onto solid Brilliance™ Salmonella agar (Thermo Scientific) and incubated at 37 °C over night. Grown bacteria were identified by Maldi-TOF-Mass-Spectrometry (Thermo Fisher Scientific, Wesel, Germany and Bruker Daltonik, Bremen, Germany) and if necessary by additional biochemical tests.

Electron microscopy was performed using the pop-off technique^62^ with a transmission electron microscope (EM10C, Carl Zeiss AG, Oberkochen, Germany). The pop-off technique allows an ultrastructural analysis of histologically conspicuous structures by directly embedding the tissue region of interest^63–65^.

MSwab swabs (Copan Italia SpA, Brescia, Italy) were used for direct investigation of the vaginal and cervical tissue. Cervical (n = 2) and vaginal swabs (n = 2) of two individuals, taken during necropsies as well as from frozen samples of the reproductive tract, were tested for the presence of *Halichoerus grypus* DNA. From each swab sample, two DNA isolations were performed and tested twice in different PCR preparations. The swab was stroked across the tissue of the suspected organs and subsequently submerged into the associated medium (1 mL MSwab medium contains Tris HCl, EDTA, TRIS Base, Dimethyl Sulfoxide (DMSO), Bovine Serum Albumin and distilled water).

The total cellular DNA was isolated using the Maxwell RSC Blood DNA Kit (Promega GmbH, Mannheim, Germany). Briefly, 300 µL MSwab medium was mixed with 20 µL proteinase K and 300 µL lysis buffer (Promega GmbH). After applying 300 µL in the kit cartridge, incubation at 56 °C followed for 30 min. The DNA was eluted with 50 µL provided elution buffer.

In addition, formalin fixed paraffin-embedded (FFPE) samples (five samples from three harbour seals with intralesional spermatozoa, four samples from harbour seals of the case series and unrelated cases from the local archive, four samples from grey seals obtained from the local archive; total n = 13) were also investigated for the presence of *Halichoerus grypus* DNA. The DNA of (FFPE) samples was isolated using the QIAmp DNA FFPE Tissue Kit (Qiagen, Hilden, Germany, Cat. No. /ID: 56,404) in accordance with the manufacturer’s protocol.

As there is a close genetic relation between the mitochondrial sequences of *Halichoerus grypus* (ACCESSION X72004) and *Phoca vitulina* (ACCESSION X63726), a comparison of the two mitochondrial sequences of both animals was performed using the Basic Local Alignment Search Tool (BLAST) of the National Center for Biotechnology Information (NCBI). A specific primer set for *Halichoerus grypus* targeting a region between the NADH5 gene and the NADH6 gene of the mtDNA genome was designed using Primer Express V2 (Applied Biosystems, Deutschland GmbH, Darmstadt, Germany).

The primer set was selected for its position within the appropriate mitochondrial sequences of *Halichoerus grypus* covering mismatches of bases, which occur in otherwise equal sequences of *Phoca vitulina*. Each primer includes three mismatches at binding sites of the 3′-end of each primer (bold letters). The primers were synthesised by Eurofins Genomics Germany GmbH, Ebersberg, Germany, and had the sequence primer Hg-3-F 5 ‘-ATCAGTCACTATCTCCAA**T**CAGAA**A**GG**T**—‘3 (primer position on acc. X72004 14,377) and the primer Hg-3-R 5 ‘-TTGTGACTGGGTGATC**T**CTT**T**TTA**T**T—‘3 (primer position on acc. X72004 14,530). The amplification size of the PCR product was 153 bp.

**Figure 6:**
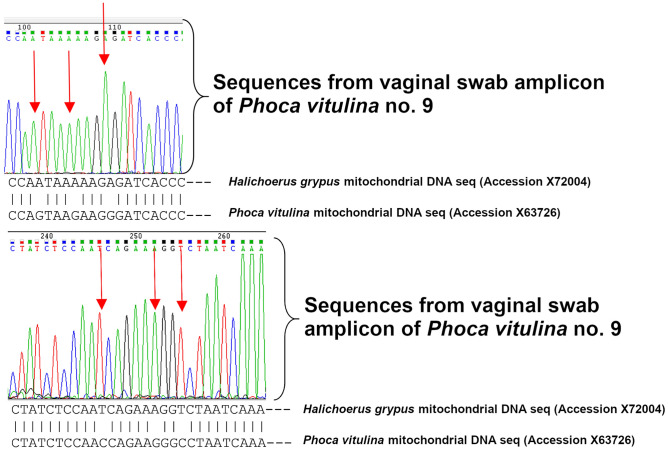
Sequence analysis of two regions of the PCR-amplicon of the *Halichoerus grypus*-DNA obtained from vaginal swab of seal no. 9.

The optimized 25μL PCR mixture for the PCR assay had a final concentration of 0.4 μM (1µL) of the primers (Hg-3-F and Hg-3-R), 12,5μL of the 2xPCR MasterMix (Roche Diagnostics GmbH, Mannheim, Germany), 8µL PCR gradient water and a 2.5μL aliquot of the DNA sample. Thermal cycling conditions comprised a Hot Start DNA polymerase activation at 95 °C for 5 min, 40 cycles of denaturation at 95 °C for 10 s, an annealing at 60 °C for 10 s and an extension 72 °C for 10 s. For the quality assurance of the specificity of the PCR reactions, a reference purified isolated DNA of *Halichoerus grypus* (~ 50 ng/µL) served as a positive PCR control. The negative control was a purified reference *Phoca vitulina* DNA (~ 30 ng/µL); a non-template control was used as master mix control. The amplicons were determined by electrophoresis of 10 μL of the reaction products in a 2% agarose gel (Peqlab Biotechnologie GmbH, Erlangen, Germany), with Trisacetate-electrophoresis buffer (TAE, pH 7.8) and a 100-bp DNA ladder (Roche Diagnostics GmbH) as molecular marker (Fig. 5). The PCR product was sent to Eurofins Genomics GmbH, Enbersberg, Germany, for sequencing and the obtained sequences were compared with *Halichoerus grypus* and *Phoca vitulina* reference sequences from NCBI (Fig. 6).

## Supplementary information

Supplementary file1
