# Supplementary material for: Male grey seal commits fatal sexual interaction with adult female harbour seals in the German Wadden Sea
Source: Sci Rep. 2020 Aug 13;10:13679. doi: 10.1038/s41598-020-69986-w (PMC7426965; doi:10.1038/s41598-020-69986-w)
Supplement: Supplementary file 1 — Supplementary file1 [file 41598_2020_69986_MOESM1_ESM.pdf]

Male grey seal under suspicion: fatal sexual interaction with adult, female harbour seals in the German Wadden Sea

Simon Rohner[1†], Kirsten Hülskötter[2†], Stephanie Gross[1], Peter Wohlsein[2], Amir Abdulmawjood[3], Madeleine Plötz[3], Jutta Verspohl [4], Ludwig Haas[5+], Ursula Siebert[1*]

[1]Institute for Terrestrial and Aquatic Wildlife Research, University of Veterinary Medicine Hannover, Foundation, Schleswig-Holstein, Germany

[2]Department of Pathology, University of Veterinary Medicine Hannover, Foundation, Germany

[3]Institute for Food Quality and Food Safety, Research Center for Emerging Infections and Zoonoses, University of Veterinary Medicine Hannover, Foundation, Hannover, Germany

[4]Institute for Microbiology, University of Veterinary Medicine Hannover, Foundation, Germany

[5]Institute of Virology, Department of Infectious Diseases, University of Veterinary Medicine Hannover, Foundation, Germany

[†]both authors contributed equally

*Corresponding author

+Ludwig Haas unfortunately died recently

Email addresses:

SR: Simon.rohner@tiho-hannover.de

KH: Kirsten.huelskoetter@tiho-hannover.de

SG: Stephanie.gross@tiho-hannover.de

PW: Peter.wohlsein@tiho-hannover.de

AA: Amir.abdulmawjood@tiho-hannover.de

MP: Madeleine.ploetz@tiho-hannover.de

JV: Jutta.verspohl@tiho-hannover.de

US: Ursula.siebert@tiho-hannover.de Tel.: +49 511 856 8158

Supplementary materials

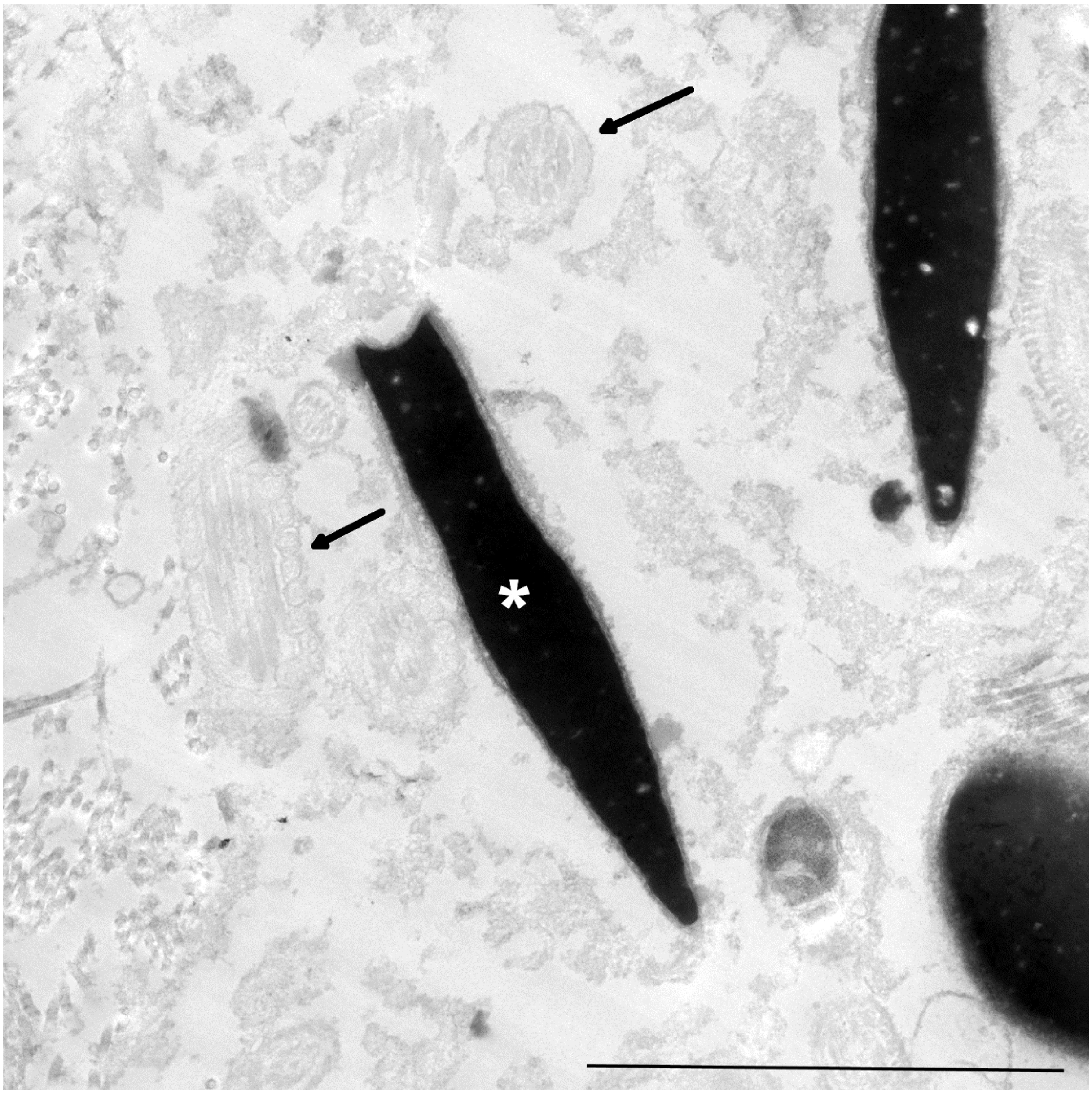

Suppl. fig. 1: Transmission electron microscopy of the vagina (no. 11). Spermatozoal heads with electron dense chromatin (*), indistinct middle pieces (→) and remnants of tails. TEM. Bar = 2500 nm.
